# Supplementary material for: Early Detection and Dynamic Changes of Circulating Tumor Cells in Transgenic NeuN Transgenic (NTTg) Mice with Spontaneous Breast Tumor Development
Source: Cancers (Basel). 2021 Jun 30;13(13):3294. doi: 10.3390/cancers13133294 (PMC8267737; doi:10.3390/cancers13133294)
Supplement: Supplementary file 1 [file cancers-13-03294-s001.zip › cancers-1156945-supplementary_org.pdf]

# Supplementary Materials:

# Early Detection and Dynamic Change of Circulating Tumor Cells in Transgenic NeuN Transgenic (NTTg) Mouse with Spontaneous Breast Tumor Development

**Wen-Sy Tsai, Tsung-Fu Hung, Jia-Yang Chen, Shu-Huan Huang and Ying-Chih Chang**

### Lumpectomy for Primary Culture of Tumor Cell and Pathology Examination

Lumpectomy was performed on the mouse under anesthesia. After disinfection of the skin, incision of skin and dissection of tumor from surrounding tissue was performed by mosquito clamps and electric cauterization of bleeding vessels. The operative wound was then cleaned with the solution contained 100 mg/mL cephazolin after resection of the tumor. The wound was closed by 5-0 nylon sutures and covered by gauze with gentamycin and metronidazole ointment. The mice were recovered on the heating pad with an oxygen supply. The dissected tissues were fixed by formaldehyde and paraffin embedded for tissue sections. The Hematoxylin-Eosin stain was used for morphological and pathological determination by professional doctors.

The primary culture of tumor cells was performed according to the previous study [1]. Briefly, the tumor tissue was chopped into 3-4 mm pieces, washed by phosphate-buffered saline solution (PBS), and then digested by 0.25% trypsin-ethylenediaminetetraacetic acid (Trypsin-EDTA (1X), phenol red, Gibco) at 4 °C overnight for 16 h. The trypsin solution was then removed, and the tissue was incubated in the residual trypsin at 37 °C for 30 min. DMEM (Dulbecco modified Eagle medium) complete medium (supplemented with 5% fetal calf serum, penicillin-streptomycin, and sodium pyruvate) was then added, and the tissue was pipetted up and down to disperse. Large pieces of undispersed tissue were allowed to settle down, and the cell suspension was pipette away from these pieces. The cells were counted and seeded into tissue culture flasks for culture in DMEM completed medium. Several rounds of differential trypsinization were performed to deplete the fibroblasts during culture to enrich more adherent epithelial cells.

The primary cultured tumor cells were trypsinized and placed on a porous polycarbonate membrane (2 ◎m pore in diameter). Between each process, the cells on the membrane were washed 3 times by PBS for 5 min. The cells were then fixed by 4% paraformaldehyde for 10 min and blocked by 5% bovine serum albumin (BSA, Sigma-Aldrich) in PBS for 1 hour at ambient temperature. After the blocking step, the cells were stained by the primary antibodies against CD45 (ab25052, Abcam), HER2 (ab2428, Abcam), PanCK (BP5069, Acris) at 4 °C overnight and incubated in secondary antibodies of Alexa488 (Invitrogen), Alexa568 (Invitrogen), and Cy5 (ab102372, Abcam) at ambient temperature undercoverfor 1 hour, respectively. DAPI(4′,6-diamidine-2′-phenylindole dihydrochloride, ThermoFisher) was used for nucleus staining. The filter membrane containing the cells was fixed with a mounting medium and covered by a coverslip. For pathological analysis of a tumor, the tumor tissue was trimmed and embedded into CBT and stored at -80 °C. A cryosection was performed within 1 week by rotary microtome at 3 mm thickness. The staining protocol was as described in the previous section.

### CTC Captured and Immunostaining-based Enumeration by using mCMx Chip

The peripheral blood derived from the NTTg mice was collected by retro-orbital venipuncture with a heparin-coated capillary tube and diluted by EDTA-PBS for mCMx pro-

cessing. Subsequently, PBS buffer was used for rinse and wash the entire mCMx microfluidic system. After target cell isolation and subsequent release, the eluent was collected onto the porous membrane for following staining. The cells were stained with guinea-pig anti-mouse pan-cytokeratin (PanCK, BP5069, Acris), and rabbit anti-mouse HER2 (ab2428, Abcam) antibodies followed with DAPI (Sigma-Aldrich) nuclear staining. Secondary antibodies including, goat-anti-rabbit AlexaFluor568 (Invitrogen), and goat-anti-guinea-pig Cy5 (ab102372, Abcam) were used for immunofluorescence staining. The staining of HER2 and PanCK were showed in green and red color, respectively. The image was photographed by Leica DMI600 B, 10x objective, 10x eyepieces with Hamamatsu ORCA-03G CCD camera, and Metamorph image system.

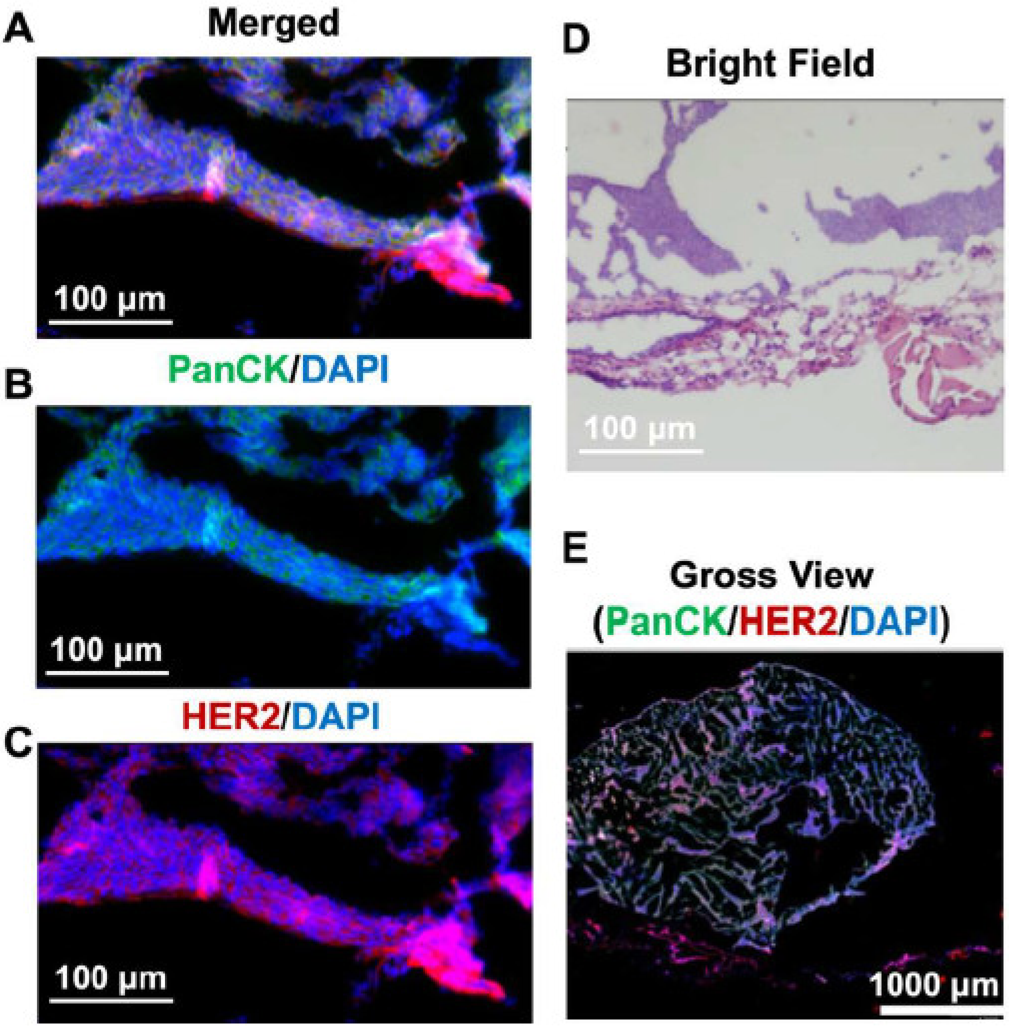


**Figure S1.** Serial Immunofluorescence stained sections of the NTTg-derived primary tumor. (A) Overlay of immunofluorescence staining of the NTTg-derived primary tumor. (B) PanCK with DAPI nuclear stain on the NTTg-derived primary tumor section. (C) HER2 with DAPI nuclear stain on the NTTg-derived primary tumor section. (D) Bright field of the HE-stained on the NTTg-derived primary tumor section. (E) Gross view of the whole on the NTTg-derived primary tumor with PanCK, HER2, and DAPI staining. Green: PanCK, Red:HER2, Blue: DAPI.

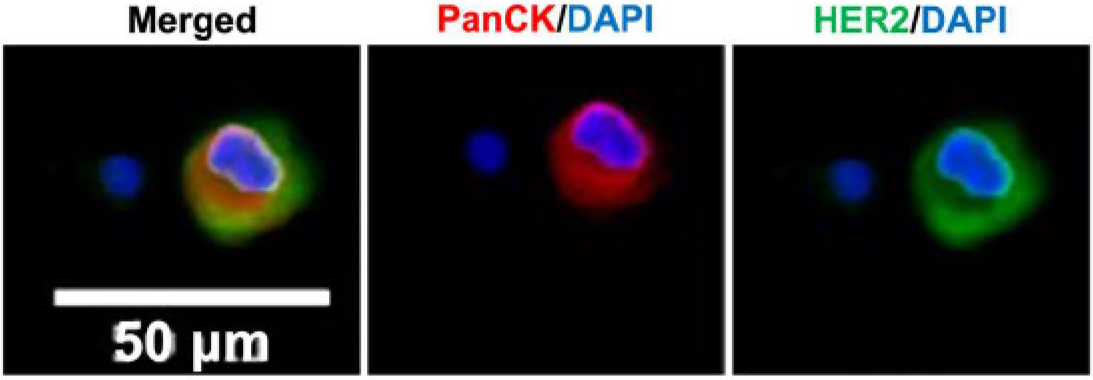


**Figure S2.** Positive stained PanCK+/HER2+/DAPI+ CTCs isolated from tumor bearing blood sample of the NTTg mice. Green: HER2, Red: PanCK, Blue: DAPI. Scale bar: 20 µm.

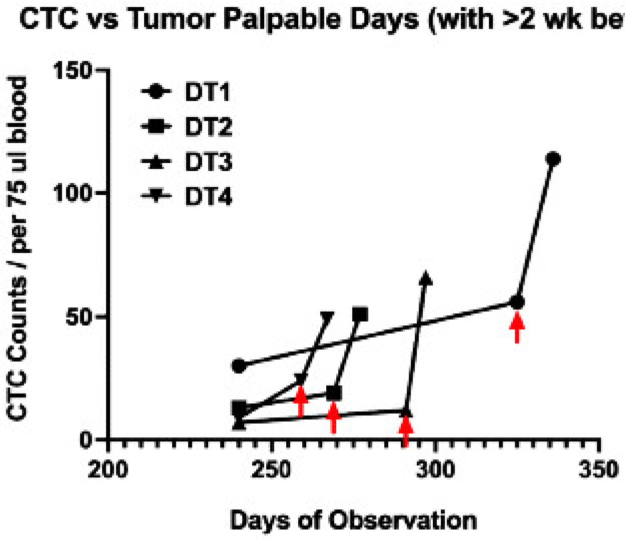


**Figure S3** The CTC counts of individual NTTg mouse models over time. The red arrows point to th day that tumor became palpable on each mouse (83 days (DT1), 28 Days (DT2), 51 days (DT3), and 17 days (DT4)).

## Supplementary References


1. Campbell, M.J.; Wollish, W.S.; Lobo, M.; Esserman, L.J. Epithelial and fibroblast cell lines derived from a spontaneous mammary carcinoma in a MMTV/neu transgenic mouse. *Vitr. Cell. Dev. Biol. Anim.* **2002**, *38*, 326–333.
